# Determining the effect of air quality on activities of daily living disability: using tracking survey data from 122 cities in China

**DOI:** 10.1186/s12889-022-13240-7

**Published:** 2022-04-26

**Authors:** Huan Liu

**Affiliations:** School of Public Administration, Zhejiang University of Finance & Economics, No. 18 Xueyuan Street, Xiasha Higher Education Park, Hang Zhou, 310018 Zhejiang China

**Keywords:** Air quality, ADL disability, CHARLS, Pollutants, Ordered logit

## Abstract

**Background:**

Current research on activities of daily living (ADLs) disability has mostly focused on the analysis of demographic characteristics, while research on the microcharacteristics of individuals and the macroenvironment is relatively limited, and these studies solely concern the impact of air quality on individual health.

**Methods:**

This study innovatively investigated the impact of air quality on ADL disability by matching micro data of individuals from the China Health and Retirement Longitudinal Study with data of urban environmental quality from 122 cities. In this study, an ordered panel logit model was adopted for the benchmark test, and the two-stage ordered probit model with IV was used for endogenous treatment.

**Results:**

This innovative study investigated the impact of air quality on ADL disability by matching individual micro data from the China Health and Retirement Longitudinal Study with urban environmental quality data for 122 cities. The results showed that air quality significantly increased the probability of ADL disability. The positive and marginal effect of air quality on moderate and mild disability was higher. Generally, the marginal effect of air quality on residents’ health was negative. In terms of group heterogeneity, the ADL disability of individuals aged over 60 years, those in the high Gross Domestic Product (GDP) group, females, and those in the nonpilot long-term care insurance group was more affected by air quality, and the interaction between air quality and serious illness showed that the deterioration of air quality exacerbated the ADL disability caused by serious illness; that is, the moderating effect was significant.

**Conclusions:**

According to the equilibrium condition of the individual health production function, the ADL disability caused by a 1% improvement in air quality is equivalent to the ADL disability caused by an 89.9652% reduction in serious illness, indicating that the effect of improved air quality is difficult to replace by any other method. Therefore, good air quality can not only reduce ADL disability directly but also reduce serious illness indirectly, which is equivalent to the reduction of ADL disability. This is called the health impact.

## Introduction

Since the beginning of the twenty-first century, the rapid development of China’s economy has been accompanied by a considerable increase in Gross Domestic Product (GDP). The per capita GDP reached 72,371 yuan in 2020 [1]. Consequently, the living standards of residents have also significantly improved. However, air pollution caused by economic development in all parts of China also increased, negatively impacting the health of the Chinese people. Outdoor air pollution was included in the list of carcinogens published by the International Agency for Research on Cancer of the World Health Organization in 2017 because dense particulate matter in the air can cause a significant impact on human health [2]. Both in China and globally, environmental protection is increasingly becoming a major issue for society as a whole. In 2017, Comrade Xi Jinping prioritized protecting the environment and maintaining harmony between man and nature in the 19th major report of the committee party [3]. Currently, it is necessary to adhere to the development concept of “Green mountains and green waters are golden mountains and silver mountains” and follow the basic state policy of conserving resources and protecting the environment. Individuals recognize that environmental protection is related to their fundamental wellbeing. Therefore, the study of air quality as it relates to environmental protection has important theoretical and practical significance.

Furthermore, from the perspective of China’s ageing population, disability has increasingly become a major livelihood problem. Existing research on the disabled population mostly focuses on the analysis of public and social policies or is conducted from a medical perspective. These studies include the analysis of the effectiveness of long-term care insurance (LTCI) for the disabled population [4, 5]; the analysis of the social characteristics of disabled people and their average life expectancy [6–8]; and the analysis of the internal physical changes that occur due to disability using the disability evaluation scale [9, 10].. On the other hand, from the perspective of air quality, the study of residents’ disability is rare. However, existing research has shown that changes in air quality have an important impact on human health. The change in individual health, especially the impact of serious illness, is usually the key factor or even the only direct factor for the impairment in activities of daily living (ADLs). Therefore, to address these gaps in the research, this study aimed to assess the impact of air quality on ADL disability in Chinese residents. The findings discussed here will provide evidence for prioritizing government programs to deal with the issues of ADL disability.

## Literature review

There is abundant research concerning the impact of air pollution on health. From the macro perspective of health impact, Usmani et al. clearly gave the definition of air pollution, the motivation to study air pollution, and the impact and source of air pollution and climate change [11]. Han et al. provided a new measurement standard for evaluating global health inequality from the perspective of climate change and air pollution control efficiency (abbreviated as APCI) [12]. In general, air pollution is closely related to the national or regional average health level. If emission reduction efforts are shared by all countries, in all scenarios, the benefits of common health would far exceed the political costs [13]. Based on the exposure response function of epidemiology, it was revealed that the impact of future temperature changes on citizens’ health is more significant than the change in air pollutant concentration [14]. Among the environmental indicators, cultivated land is the indicator that shows the greatest impact on health and wealth in the next 10 years, while air pollution has the least impact on health and wealth for low-income countries [15]. However, it was found that environmental and air pollution impose a great threat on the health and wealth of residents in low-income countries. Moreover, there are significant differences in the effects of different pollutants. From the perspective of the impact pathway of pollution, NO_2_ and O_3_ are more important, and their AR (added health risk) decreases significantly in urban areas with crowded traffic, but no significant change in AR was found in other areas with low urbanization [16].

Among the research on individual health impacts, on the one hand, air pollution indeed has an impact on individual health [17–21]; on the other hand, it also affects potential medical consumption [22, 23]. In detail, (1) as one of the primary outcomes of the impact of air pollution, the death rate of respiratory diseases is increasing significantly [24], and this economic cost even exceeds the economic benefits. As a result, production efficiency decreased. For instance, based on the HAQI (health risk-based AQI), it was estimated that 20% of the population in the study area was exposed to polluted air. The total mortality rates caused by PM_10_, PM_2.5_, SO_2_, O_3_, NO_2_, and CO were 3.00, 1.02, 1.00, 4.22, 1.57, and 0.95%, respectively [25]. In addition, inhalable particles in air pollutants affect individual health mainly in two ways: one is the short-term effect on the human respiratory tract, which can cause respiratory tract infection, chronic obstructive pulmonary disease, lung cancer, and other respiratory diseases [26–29]; the other is the long-term impact on the respiratory tract that involves the triggering of the inflammatory cascade through local inflammatory factors, ultimately leading to a significant increase in the risk of cardiovascular and nervous system diseases [30–34]. As the research revealed, when PM_10_ and O_3_ in air pollutants increase by 10 μg/m3 and 10 ppb, the number of visitors to respiratory hospitals in 1 day will increase by 10.39 and 10.93%, respectively. This would bring about additional medical expenses of $67 million and $70 million, respectively [35]. Furthermore, the health effects of air pollution vary under different socioeconomic statuses. For example, self-rated air pollution has the greatest impact on the self-rated health of low socioeconomic groups, while with the improvement of socioeconomic status, the impact of self-rated air pollution on self-rated health decreases [36]. (2) Air pollution indirectly affects residents’ medical consumption. Sun et al. demonstrated that air pollution is also the main factor that influences residents’ expenditures on health management [37]. Theoretically, air pollution affects health mainly in two ways: first, the reduction in sleep time caused by ambient air pollution is not conducive to health; second, people spend more time on sedentary activities to avoid exposure to air pollution, which will indirectly lead to an increase in personal medical expenditure [38]. Additionally, from the empirical results, air pollution will lead to a significant increase in medical expenses, hospitalization expenses and extrabudgetary expenses [38]. For example, Liu et al. estimated age- and cause-specific premature deaths and quantified related health damage with the measurement of the age-adjusted value of statistical life (VSL). Their results suggest that while premature deaths fell as a result of China’s clean air actions, the health costs of air pollution remained high [39].

Most of the existing studies on residents’ ADLs are based on the micro viewpoints of individual disease risk. For example, in ADL disability assessment, based on the diagnosis rate of major diseases, individual disease risks are defined by establishing the relevant Disability Assessment Scale [5, 6]. However, even in countries or regions with long-term implementation of health care insurance, the impact of air pollution on residents’ ADL disability has rarely been investigated, neither in practice nor in theory. This also illustrates the major significance of this study. Current research in this field focuses on the factors that influence the population’s health via urban green spaces, the ecological environment and air quality. The findings from such studies show that the deterioration of the ecological environment negatively impacts human health. However, there are some gaps in the existing research. First, although there are relatively abundant studies on the impact of the ecological environment on individual health, the majority of these focus on direct health effects, ignoring the cumulative indirect effects of changes in environmental quality. Furthermore, these studies focus only on medical expenses. Second, in the measurement of air quality, the traditional air pollution index (API) or the concentration of a single pollutant are often used for testing. Although it is suitable to investigate the impact of a single pollutant, for estimates that are closer to the real-world impact, testing should include a comprehensive list of pollutants. Third, existing studies mainly focus on the impact of air quality on individual health without fully considering internal transmission mechanisms through which air quality affects health. To address these gaps, this study focused on the following points. First, we investigated the indirect impact of air pollution by assessing the decline in residents’ basic activities of daily living (ADLs). Second, sulfur dioxide (SO_2_), nitrogen dioxide (NO_2_) and inhalable particles (PM_10_) were included as proxy variables, and China Health and Retirement Longitudinal Study (CHARLS) data from 2015 and 2018 were matched with macro regional air quality data to construct panel data. Heterogeneity analysis and endogenous problem processing were used to ensure the reliability of the test results. The air quality index (AQI) was introduced to investigate the robustness of the results, considering the heterogeneity of a single air quality index and the overall impact. Third, by constructing the health production function, we investigated the substitution effect of air quality and serious illness on individual ADL disability and tested the transmission mechanism of air quality impacting individual ADL disability.

## Methods

### Theoretical hypothesis: impact of air quality on health

**Fig. 1:**
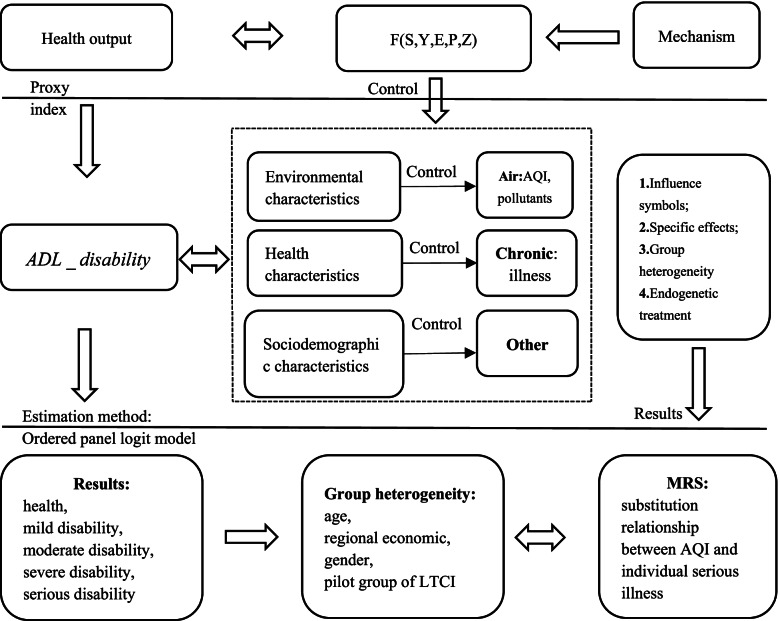
Theoretical mechanism of the impact of air pollution on Residents’ ADL disability

The health demand model was first proposed by Grossman [40], and the health production function, which is the core of the supply model, is derived from it. The health production function can be divided into macro and micro parts, which are interrelated. Among them, the microhealth production function emphasizes the relationship between family- or individual-level medical and health input and individual health output through macro policy intervention [41, 42]. The macrohealth production function considers the overall output effect of national health from the perspective of macroeconomics, government health expenditure, and medical insurance [43]. This study investigated air quality effects from a macro perspective by analysing the macro health production function. The theoretical mechanism of the impact of air pollution on residents’ health is shown in Fig. 1.

Based on Grossman’s health demand model, Filmer et al. [44] constructed a macro health production function model. Health needs are formed by the correlation between health and related factors that improve health. The core of the health production function is composed of output factors and health inputs. Due to the relevant hypothesis bias in the micro field, there is an estimation bias in the analysis of medical and health policy inputs and outputs using the perfect competition market model. Therefore, more nonendogenous factors must be explained. When health economists use the general production function theory, combined with health characteristics, they put forward that in the process of maintaining or improving health, the input and output of medical and health resources are included in the basic health production function. Therefore, the general health production function can be expressed as:

1 $$H=F\left(S,\kern0.5em Y,\kern0.5em E,\kern0.5em P,\kern0.5em Z\right)$$

Equation (1) is the national health level at a certain time point, where *S* represents the input of social factors, *Y* is the input of economic variables, *E* is the input of educational variables, *P* is the input of medical and health policies and *Z* is the social health investment. However, the existing health production function does not consider the impact of the natural environment or air quality. Therefore, this study used individual ADL disability as a proxy for health variables and assumed that ADL disability is influenced by sociodemographic, regional environmental and individual health characteristics [45]. Here, sociodemographic characteristics include gender, age, household-registered marital status, etc. Regional environmental characteristics include regional financial expenditure, per capita GDP, population density, sunshine duration and rainfall. Individual health characteristics include serious illness, depression and self-reported health. Therefore, the health production function can be adjusted as follows:

2 $$ADL\_ disability=F\left(R,\kern0.5em H,S\right)$$

In Eq. (2), *ADL* _ *disability* is calculated; R on the right side of the equation represents the regional environmental characteristics, H represents the individual health characteristics, and S represents the individuals’ sociodemographic characteristics. Based on existing research and the objectives of this study, air quality was considered the primary factor of ADL disability, while other influencing factors were taken as control variables. Therefore, Eq. (2) can be adjusted as follows:

3 $$ADL\_ disability=F\left(\mathrm{A}i\mathrm{r}, Chronic, Other\right)$$

The pilot for China’s LTCI showed that the most important cause of disability for most severely disabled persons was the occurrence of serious illness [5]. Therefore, this study considered the rate of serious illness (i.e., diagnosis rate of serious illness) as an important regulatory index to investigate the detrimental effect of air quality on individual ADL. The *Chronic* on the right of Eq. (3) is the serious illness rate. In addition, after controlling for other factors, we can further investigate the substitution relationship between air quality and serious illness, which can be derived from Eq. (3). When the individual ADL disability remains unchanged, it should be equal to 0, that is:

4 $$dADL\_ disability=\frac{\partial ADL\_ disability}{\mathrm{\partial Air}}\bullet dADL\_ disability+\frac{\partial ADL\_ disability}{\partial Chronic}\bullet d Chronic= 0$$

Then, the marginal substitution rate between air quality and residents’ serious illnesses can be:

5 $${\left. MRS\right|}_{Air}=\frac{d\mathrm{C} hronic}{d\mathrm{Air}}=-\frac{\partial ADL\_ disability/\mathrm{\partial Air}}{\partial ADL\_ disability/\mathrm{\partial C} hronic}$$

**Fig. 2:**
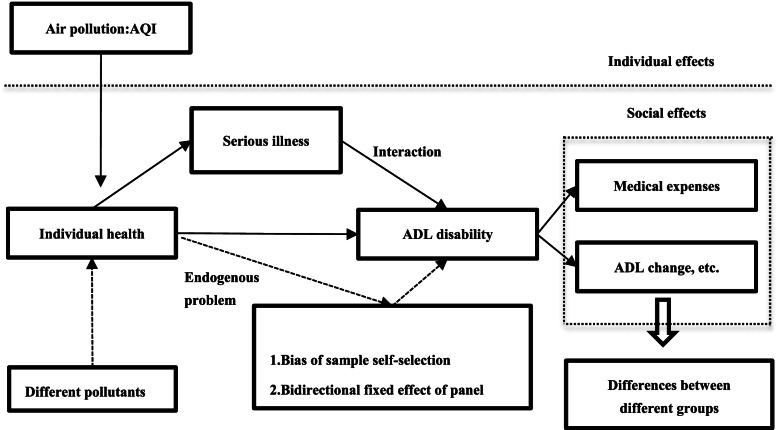
Effect of air pollution on the ADL disability of residents

Equation (5) shows the substitution relationship between air quality and individual serious illness under the condition of constant ADL disability. Therefore, the reduction in individual serious illness by a one-unit improvement in air quality represents the health impact of air quality, which is measured by the changes in ADL disability due to air quality. The empirical method testing the impact of air pollution on residents’ health is shown in Fig. 2.

### Test model

Based on the above theoretical analyses of the health impact of air quality, this study further constructed an empirical test model. Considering that the core explanatory variable of this study was residents’ ADL disability, we classified ADL disability. Please refer to the definitions of core explanatory variables and classifications in the data section for specific explanations. This implied that the traditional OLS estimation would result in bias; therefore, the ordered panel logit model was selected for the test:

6 $$ADL\_{\mathrm{disability}}_{\mathrm{ijt}}=F\left(\alpha \ln \kern0.5em \mathrm{A}i{\mathrm{r}}_{\mathrm{jt}}+\beta {Chronic}_{\mathrm{ijt}}+\kappa {H}_{ijt}+\chi {R}_{jt}+\varphi {S}_{ijt}+{\lambda}_i+{\delta}_j+{\eta}_t+{\varepsilon}_{ijt}\right)$$

In Eq. (6), *ADL* _ *disability* represents the ADL disability of individual *i* living in city *j* in year *t*, which is the primary explained variable of this study; *Air*_*jt*_ on the right side of the equation represents the air quality of city *j* in year *t*, which is another primary explanatory variable of this study. In this study, SO_2_, NO_2_, and PM_10_ in the API were selected as proxies of air quality, and the AQI was selected for the robustness test. In the data processing step, to avoid the influence of nondimensional values, logarithmic processing was used. *H*_*ijt*_ represents individual health characteristics, including individual serious illness rate, self-reported health and physical pain. *R*_*jt*_ represents the environmental characteristics of *j* city in *t* year, including annual rainfall and annual sunshine duration. *S* indicates sociodemographic characteristics such as gender, age, marital status, etc. Since the panel logit model only provides the test results of random effects, to ensure reliable results, the individual effect, regional effect, and year effect were controlled simultaneously in the model, which were *λ*_*i*_, *δ*_*j*_ and *η*_*t*_ in Eq. (6), respectively. *ε*_*ijt*_ represents random error. Furthermore, the health production function of Eq. (6) is nonlinear; therefore, it satisfies the following conditions:

7 $$F\left( ADL\_{Disability}_{ijt}^{\kern1.75em \ast}\right)=\left\{\begin{array}{c}1, ADL\_{Disability}_{ijt}^{\kern1.75em \ast}\le {r}_0\\ {}2,{r}_0< ADL\_{Disability}_{ijt}^{\kern1.75em \ast}\le {r}_1\\ {}3,{r}_1< ADL\_{Disability}_{ijt}^{\kern1.75em \ast}\le {r}_2\\ {}J,{r}_{J-1}\le ADL\_{Disability}_{ijt}^{\kern1.75em \ast}\end{array}\right.$$

where *ADL* _ *disability*_*ijt*_^∗^ is the unobservable continuous variable of *ADL* _ *disability*_*ijt*_, which is the latent variable and satisfies the assumption of linearity. In Eq. (7), *r*_0_, *r*_1_, *r*_2_... denote the parameters to be estimated. To keep the ADL disability of residents unchanged, we can investigate how serious illness was impacted when air quality deteriorates. Based on the above analysis, the marginal substitution rate between air quality and serious illness can be adjusted to Eq. (8) based on Eq. (5), where |*α*/*β*| is the substitution rate between serious illness and air quality, as given below:

8 $${\left. MRS\right|}_{Air}=\frac{\partial ADL\_ disability/\mathrm{\partial Air}}{\partial ADL\_ disability/\mathrm{\partial C} hronic}=-\frac{\alpha }{\beta}\times \frac{Chronic}{Air}$$

Considering the characteristics of the health production function, we should determine the substitution relationship between air quality and serious illness and how to improve air quality and reduce serious illness at the same time when the overall ADL disability is reduced. This is for determining the scale effect of the health production function and verifying the marginal effect of each variable in the real test, which will be discussed later.

## Data

### Individual ADL disability data

The individual micro data of this study were obtained from the CHARLS surveys of 2015 and 2018. The data that support the findings of this study are openly available at the following URL/DOI: http://charls.pku.edu.cn/. In this dataset, there were 12,520 participants from 2015 and 13,358 from 2018. By controlling for individual and time effects, as well as for sociodemographic characteristics of the population and the macro characteristics of the city, the reliability and accuracy of the estimated effect of air quality on individual ADLs were improved.

The core explanatory variable for the analysis was the ADL disability of residents, and the specific indicators were defined as follows: ADLs were determined based on the question “whether you have difficulties in dressing, bathing, eating, getting up and out of bed, going to the toilet, controlling defecation and defecation”. The score for this question was based on the selection of options from 1-no difficulty, 2-difficulty but still can be completed, 3-difficulty and need help, and 4-unable to complete. In total, six basic self-care ability indicators were used, and the total score ranged from 6 to 24. Based on the existing classification of disability, ADL disability was divided into five levels: serious disability, severe disability, moderate disability, mild disability, and healthy [6]. Through data processing, a total score of 6 was recorded as 5, which represented “healthy”; a score of 7–9 was defined as 4, indicating a mild disability; a score of 10–14 was recorded as 3, indicating moderate disability; a score of 15–20 was defined as 2, which indicated severe disability; and a score of 21–24 was 1, which indicated serious disability. Therefore, a higher ADL disability score indicated a lower degree of ADLs.

The statistics of the probability of ADL disability are presented in Table 1. As shown in Table 1, the rates of serious disability, severe disability and moderate disability increased from 2015 to 2018. The proportion of people with severe and mild ADL disability in the total population increased from 6.29 to 7.93%, but the proportion was still lower than that with mild disability. In addition, the proportion of the healthy population increased by a small degree during this period.

**Table 1:** Probability statistics of ADL disability

ADL disability	2015		2018	
	Relative frequency	Frequency (%)	Relative frequency	Frequency (%)
Serious disability	23	0.18	92	0.69
Severe disability	101	0.81	174	1.30
Moderate disability	664	5.30	794	5.94
Mild disability	2664	21.28	2482	18.58
Healthy	9068	72.43	9816	73.48

### Air quality data

There are many measurement indicators of air pollution, such as the air quality index (AQI) and air pollution index (API). While the main pollutants in exhaust gas were mainly industrial emissions, the API indicator was not a comprehensive measure of air quality [46]. The AQI is a more comprehensive measure, and its data are released once an hour. Therefore, it is advantageous to use the annual average AQI value to investigate the impact of air quality on ADL disability [47].

### Control variables

In addition to air quality, the main factors of ADL disability include sociodemographic characteristics and other factors. The definition and statistics of the control variables in this study are shown in Table 2, including the regional natural environment, economic environment, and individual and family characteristics.

**Table 2:** Descriptive statistics of main variables

Variable	Definition	2015 (12520)		2018 (13358)	
		Mean	SD	Mean	SD
ADL disability	1 ~ 5; higher score indicated lower ADL disability	4.651	0.513	4.636	0.595
SO_2_	SO_2_ content in air (μg /m^3^)	27.53	17.96	16.26	10.58
NO_2_	NO_2_ content in air (μg /m^3^)	32.74	11.62	39.23	15.93
PM_10_	PM_10_ content in air (μg /m^3^)	94.38	40.94	89.74	40.32
AQI	Dimensionless air quality; greater value indicated poorer quality	85.76	25.79	72.14	16.55
Fiscal expenditure	Total annual financial expenditure of the region (million yuan)	544.9	729.6	688.0	1030
Sunshine duration	Total sunshine duration in the whole year, (hour)	1814	469.0	1903	354.4
Rainfall	Annual total rainfall (mm)	1067	624.8	997.3	441.2
Per capita GDP	Annual regional GDP to population ratio, (yuan / person)	49,467	34,418	56,468	35,992
Population density	Annual area to population ratio (Person / m^2^)	490.1	479.4	492.6	473.1
Average temperature	Annual average temperature (centigrade)	15.24	3.867	15.08	3.926
GDP growth	Regional GDP growth compared with the previous year	8.078	2.081	7.054	1.823
Green space coverage	Ratio of green area to total area (in built up area)	39.54	9.130	39.96	5.022
Relative humidity	Percentage of water vapor pressure in air to saturated vapor pressure at the same temperature	64.65	12.39	65.03	10.64
Household register	Urban = 1, rural = 0	0.401	0.490	0.405	0.491
Income	1 ~ 5 respectively represent high income, middle-high-income, middle income, lower-middle-income and low income	2.605	0.783	2.754	0.803
Basic medical insurance	Enjoying basic medical insurance = 1, no = 0	0.945	0.137	0.971	0.168
Marital status	Widowed = 1, no = 0	0.103	0.304	0.125	0.330
Serious illness	Number of serious illnesses diagnosed; higher value indicates a greater number of illnesses	0.0294	0.286	0.724	1.052
Depression	1–4; higher score indicates more severe depression	2.468	0.740	2.275	0.783
Self-reported health	1 ~ 5; higher value indicates better health	2.955	0.721	2.946	0.986
Body disability	0–5; higher score indicates more severe body disability	0.154	0.444	0.145	0.445
Physical pain	1–5; higher score indicates more severe pain	1.705	0.456	2.159	1.267
Age	Actual age of the individual in the survey year	59.14	10.32	58.74	10.32
Gender	Male = 1, female = 0	0.478	0.500	0.474	0.499
Education level	1–11 respectively represent No formal education (illiterate),Did not finish primary school, Sishu/home school, Elementary school, Middle school, High school, Vocational school, Two−/Three-Year College/Associate degree, Four-Year College/Bachelor’s degree, Master’s degree, Doctoral degree/Ph.D.	3.390	1.001	3.477	1.935

*Abbreviations*: *ADL* Activities of Daily Living, *AQI* Air Quality Index, *SO*_2_ Sulfur Dioxide, *NO*_2_ Nitrogen Dioxide, *PM*_10_ Inhalable Particles

Note: Standard errors are in brackets; *** *p* < 0.01, ** *p* < 0.05, * *p* < 0.1. The model controls for both the year and individual effects to consider the influence of unobservable factors

Table 2 shows that the variation coefficients of ADL disability in 2015 and 2018 were 0.110 and 0.128, respectively. The degree of dispersion was small, and mild disability and health were the main parts. On the other hand, the variation coefficients of the concentrations of SO_2_, NO_2_ and PM_10_ were 0.652, 0.651, and 0.355 in 2015, respectively, and changed to 0.406, 0.434, and 0.449 in 2018. Thus, the variations in NO_2_ and PM_2_ were similar, while the dispersion of SO_2_ was relatively larger. The statistical values of the AQI in 2015 and 2018 were 85.76 and 72.14, respectively, which means that the air quality apparently improved in 2018.

## Results

### Benchmark regression

In the benchmark regression, the effects of different pollutant concentrations were tested, and the results are presented in Table 3. Models (1)–(3) are the results of the stepwise test of air pollutant concentration effects, controlled by individual and time effects, whereas Model (4) is based on the AQI. The results show that both SO_2_ and PM_10_ have significant and negative effects on ADL disability. The significance level of SO_2_ was low, whereas the results for the coefficient of PM_10_ were more robust. In other words, higher concentrations of SO_2_ and PM_10_ in the air have brought about a higher degree of ADL disability. These results demonstrate that an increased concentration of air pollutants aggravates the degree of ADL disability and that PM_10_ plays a more important role. The results of Model (4) show that air quality has a significant and negative impact on residents’ ADL disability; the worse the air quality is, the higher the degree of residents’ ADL disability. This result proves the robustness of the results of pollutant concentrations.

**Table 3:** Impact of air quality on ADL disability: Benchmark regression

Variable	(1)	(2)	(3)	(4)
lnSO_2_	− 0.0599*(0.0355)			
lnNO_2_		−0.0017(0.0533)		
lnPM_10_			− 0.1056**(0.0510)	
lnAQI				− 0.1543**(0.0721)
Fiscal expenditure	− 0.0057(0.0276)	0.0015(0.0273)	− 0.0027(0.0274)	− 0.0018(0.0271)
Sunshine duration	− 0.0204(0.0865)	− 0.0311(0.0861)	− 0.0364(0.0864)	−0.0219(0.0857)
Rainfall	0.1173*(0.0620)	0.1353**(0.0620)	0.1015(0.0624)	0.1232**(0.0611)
Per capita GDP	0.0403(0.0330)	0.0361(0.0357)	0.0473(0.0334)	0.0344(0.0328)
Population density	0.0596***(0.023)	0.0513**(0.0238)	0.0744***(0.025)	0.0666***(0.0237)
Average temperature	−0.4412***(0.093)	−0.4373***(0.093)	− 0.4503***(0.094)	− 0.4203***(0.0925)
GDP growth	0.0139(0.0090)	0.0159*(0.0090)	0.0174*(0.0090)	0.0173*(0.0089)
Green space coverage	0.0007(0.0022)	0.0007(0.0022)	0.0005(0.0022)	0.0004(0.0022)
Relative humidity	−0.0010(0.0029)	−0.0007(0.0029)	− 0.0014(0.0029)	−0.0017(0.0029)
Household register	0.1030***(0.036)	0.1015***(0.036)	0.0968***(0.036)	0.0701*(0.0358)
Income	−0.0190(0.0223)	−0.0186(0.0223)	− 0.0200(0.0223)	−0.0191(0.0222)
Basic medical insurance	0.2187(0.1489)	0.2232(0.1488)	0.2269(0.1489)	0.2268(0.1485)
Marital status	−0.1834**(0.072)	−0.1827**(0.072)	− 0.1837**(0.0721)	−0.2654***(0.0725)
Serious illness	0.0486*(0.0271)	0.0491*(0.0271)	0.0506*(0.0271)	0.0546**(0.0270)
Depression	0.1194***(0.025)	0.1183***(0.0251)	0.1194***(0.025)	0.1513***(0.0252)
Self-reported health	0.0930***(0.020)	0.0933***(0.0198)	0.0937***(0.020)	0.0866***(0.0197)
Body disability	−0.6474***(0.049)	−0.6473***(0.049)	− 0.6472***(0.049)	−0.6378***(0.0490)
Physical pain	−0.0369*(0.0215)	−0.0375*(0.0215)	− 0.0370*(0.0215)	−0.0415*(0.0215)
Age	−0.0017(0.0020)	−0.0017(0.0020)	− 0.0017(0.0020)	−0.3707***(0.0347)
Gender	−0.3703***(0.035)	−0.3705***(0.035)	− 0.3707***(0.035)	0.0010(0.0020)
Education	−0.0350***(0.011)	−0.0350***(0.011)	− 0.0349***(0.011)	−0.0132(0.0110)
Individual / Year	Yes	Yes	Yes	Yes
|*α*/*β*|	1.2325	0.0346	2.0870	2.8260
sigma2_u	1.7866***(0.119)	1.7859***(0.119)	1.7901***(0.119)	1.7249***(0.1194)
Pseudo log likelihood	− 27,316.835	− 27,318.331	− 27,316.157	− 27,260.191
Observations	26,218	26,218	26,218	26,218

*Abbreviations*: *AQI* Air Quality Index, *SO*_2_ Sulfur Dioxide, *NO*_2_ Nitrogen Dioxide, *PM*_10_ Inhalable Particles

Note: Standard errors are in brackets; * *p* < 0.01, * *p* < 0.05, * *p* < 0.1. The pseudo log-likelihood value in the table is log pseudolikelihood

In terms of control variables, population density, annual rainfall and annual average temperature had significant effects on ADL disability. Population density and annual rainfall had positive effects: the higher the population density and annual rainfall were, the lower the degree of ADL disability. On the other hand, annual average temperature had negative effects: the higher the annual average temperature was, the higher the degree of ADL disability. Regarding individual characteristics, household registration, depression, self-reported health and serious illness had positive effects on ADL disability, but marital status, disability, physical pain, gender and education had significant and negative effects on ADL disability.

These results demonstrate that the concentration of air pollutants has a significant impact on ADL disability, and among the control variables, the basic health status of individuals is the primary factor affecting ADL disability. Moreover, by looking into the marginal substitution effect of air quality and serious illness, to maintain the level of ADL disability, the decrease in ADLs caused by a 1% increase in SO_2_, NO_2_, PM_10_ and the AQI needs to be compensated by a 1.2325, 0.0346, 2.087, and 2.826% reduction in the serious illness, respectively. The substitution relationship between air quality and other health variables can also be investigated; however, they were not of interest to this study.

### Marginal effect analysis

Based on Table 3, the marginal effect of air quality on ADL disability can be further estimated, and the results are shown in Table 4. Because the ordered logit model can only provide limited information on the signs and significance of parameters, it is necessary to estimate the marginal effect of air quality on ADL disability. When all explanatory variables are at the mean value, the influence of the exogenous explanatory variables can be expressed as Eq. (9):

9 $${\left.\frac{\partial prob\left( ADL=i/ Air\right)}{\partial Air}\right|}_{Air=\overline{Air}}\left(i=1,2,3,4,5\right)$$

**Table 4:** Marginal effect of air quality on ADL disability

ADL disability	lnSO_2_	lnNO_2_	lnPM_10_	lnAQI
Serious disability	0.00003(0.00002)	8.42e-07(0.00003)	0.00005*(0.00003)	0.00008*(0.00004)
Severe disability	0.00013*(0.0001)	3.77e-06(0.0001)	0.0002**(0.0001)	0.0003**(0.0002)
Moderate disability	0.0012*(0.0007)	0.00003(0.0010)	0.0020**(0.0010)	0.0030**(0.0014)
Mild disability	0.0045*(0.0026)	0.0001(0.0040)	0.0079**(0.0038)	0.0115**(0.0054)
Healthy	−0.0110*(0.0063)	− 0.0003(0.0098)	− 0.0194**(0.0094)	− 0.0284**(0.0133)

*Abbreviations*: *ADL* Activities of Daily Living, *SO*_2_ Sulfur Dioxide, *NO*_2_ Nitrogen Dioxide, *PM*_10_ Inhalable Particles

Note: The standard error is in brackets; *** *p* < 0.01, ** *p* < 0.05, * *p* < 0.1. The control variable results are not listed here

Table 4 shows the marginal effects of air quality on the ADL disability of residents. PM_10_ is the primary factor affecting ADL disability, and when the PM_10_ concentration is increased by 1 unit, the probability of serious disability, severe disability, moderate disability, mild disability and healthy status of residents is significantly increased by 0.005, 0.02, 0.20, 0.79 and 1.94%, respectively. The marginal effect of NO_2_ is very weak and nonsignificant. In comparison, when the SO_2_ concentration was increased by one unit, the increase in the probability of serious disability, moderate disability and mild disability was 0.013, 0.12 and 0.45%, respectively, whereas the health reduction probability was − 1.10%. From the test of the marginal effect of the AQI, the above results are robust. The marginal effect of the AQI on severe, mild severe, moderate and mild disability is positive, and the marginal effect of the AQI on moderate and mild disability is higher. If the AQI is increased by 1 unit, the probability of moderate and mild disability increases by 0.30 and 1.15%, respectively. Meanwhile, the marginal effect of the AQI on health reaches 2.84%, which means that a 1 unit increase in the AQI leads to a 2.84% decrease in the probability of residents’ health.

### Analysis of group heterogeneity

To investigate the variations in the impact of air quality on ADL disability between different groups, analysis models were stratified according to age, regional economy (GDP), gender and LTCI policy pilot. These results are shown in Table 5.

**Table 5:** Heterogeneity of ADL disability among different groups of residents affected by air quality

Grouping	Indicators	lnSO_2_	lnNO_2_	lnPM_10_	lnAQI	Observations
Age group	Under 60 years	0.0170 (0.0452)	0.0917 (0.0671)	−0.0078 (0.0653)	− 0.1352 (0.0921)	15,526
	Over 60 years old	−0.1530*** (0.0567)	−0.1069 (0.0869)	− 0.2208*** (0.0801)	−0.1531 (0.1140)	10,692
Regional economic status	Low GDP group	−0.0074 (0.0408)	0.0112 (0.0576)	−0.0771 (0.0611)	−0.1275 (0.0896)	18,952
	High GDP group	−0.2994*** (0.0907)	−0.1410 (0.1680)	− 0.3922*** (0.1316)	−0.2325 (0.1521)	7266
Gender	Male	−0.0067 (0.0494)	0.0739 (0.0739)	−0.0127 (0.0712)	−0.2606*** (0.0999)	12,225
	Female	−0.1121** (0.0503)	−0.0696 (0.0753)	− 0.1812** (0.0720)	−0.0627 (0.1038)	13,993
Long term insurance pilot	Pilot was launched	0.3958 (6.8008)	−2.5769 (44.2767)	−0.3120 (5.3616)	−2.2673 (38.9568)	419
	No pilot was conducted	−0.0597* (0.0358)	−0.0048 (0.0535)	− 0.1097** (0.0515)	−0.1475** (0.0727)	25,799

*Abbreviations*: *AQI* Air Quality Index, *SO*_2_ Sulfur Dioxide, *NO*_2_ Nitrogen Dioxide, *PM*_10_ Inhalable Particles

Note: Standard errors are in brackets; *** *p* < 0.01, ** *p* < 0.05, * *p* < 0.1. The control variable results are not listed here

Regarding age, we used the elderly population with higher ADL disability risk as the division reference; thus, those aged 60 years and above were divided from others. The results show that compared with the age group under 60 years, air quality has a significantly higher impact on ADL disability of residents over 60 years. SO_2_ and PM_10_ have a significant impact on the ADL disability of residents over 60 years. This indicates that under the same conditions, the probability of ADL disability in elderly individuals brought by air quality deterioration is higher than that of the nonelderly population. However, there was no significant difference in the effect of the AQI on ADL disability by age.

In terms of regional economy, we selected the regional economic aggregate as the grouping standard; that is, the regional GDP lower than the average GDP was the low economic group, whereas the regional GDP higher than the average GDP was assigned to the high economic group. The results showed that compared with the low economic group, air quality had a more significant and negative effect on ADL disability in the high economic group. This is probably because the areas with stronger economies tend to promote better quality of life. Areas of strong economic development also have higher population density and more urban automobile pollution and industrial pollution, thus resulting in a significantly higher impact of air quality on ADL disability. In the low-level economic development area, the situation is the opposite. However, there was no significant difference in the effect of the AQI on ADL disability of different regional economic groups.

Moreover, compared with male residents, air quality had a more significant impact on ADL disability in female residents. This is because the life expectancy of female residents is generally higher than that of male residents, and in daily life, female residents are mainly engaged in household activities. Therefore, females experience more ADL disability related to cooking fume inhalation at home than males. However, the impact of the AQI on ADL disability was more significant for male residents since in general, workers in the mining industry are mostly men. Therefore, the impact of outdoor air pollution is higher for males, which increases the probability of ADL disability.

For the LTCI pilot group, the dummy variable of the pilot policy was constructed according to the implementation time of the LTCI policy in 15 pilot cities in 2016, whereby the nontreatment group and treatment group were determined. The results show that compared with the pilot areas, the air quality in the nonpilot areas had a more significant impact on ADL disability; that is, the LTCI pilot reduced the risk of ADL disability caused by air quality and promoted the prevention or rehabilitation of ADL disability among residents.

### Analysis of the interaction between air quality and serious illness

Among the individual characteristics that affect ADL disability, serious illness was the most important factor. Previous theoretical research on LTCI shows that the disabled population is mainly affected by serious illnesses such as cerebral haemorrhage and cerebral infarction. Therefore, it is of great theoretical significance to investigate the interaction between serious illnesses and air quality. The test results of the interaction items are presented in Table 6. The interaction terms of serious illness and SO_2_ and the interaction of serious illness and NO_2_ play a significant and positive role in ADL disability, and the serious disease rate has a significant and negative effect on ADL disability. However, from Table 3, which shows the estimation results for the models without interaction items, the impact of serious illness on ADL disability was significantly positive, which is contrary to reality and theory. The results for Model (4) in Table 6 also show that the interaction terms have a positive moderating effect but are not significant.

**Table 6:** Estimation of the effects of the interaction between air quality and serious illness on ADL disability

Variable	(1)	(2)	(3)	(4)
lnSO_2_	−0.0882**(0.0367)			
lnNO_2_		−0.0538(0.0555)		
lnPM_10_			−0.1246**(0.0530)	
lnAQI				−0.1548**(0.0724)
Serious illness	−0.2328*(0.1317)	−0.4058**(0.2038)	− 0.1697(0.2315)	0.0126(0.0442)
X × lnSO_2_/ X × lnNO_2_/ X × lnPM_10_/ X × lnAQI	0.1043**(0.0478)	0.1266**(0.0557)	0.0496(0.0517)	0.0225(0.0185)
Individual / Year	Yes	Yes	Yes	Yes
/sigma2_u	1.7819***(0.1183)	1.7848***(0.1186)	1.7891***(0.1186)	1.7806***(0.1183)
Pseudo log likelihood	−27,313.284	−27,314.254	− 27,315.495	−27,315.248
Observations	26,218	26,218	26,218	26,218

*Abbreviations*: *AQI* Air Quality Index, *SO*_2_ Sulfur Dioxide, *NO*_2_ Nitrogen Dioxide, *PM*_10_ Inhalable Particles

Note: Standard errors are in brackets; *** *p* < 0.01, ** *p* < 0.05, * *p* < 0.1. The control variable results are not listed here

X is the rate of serious illness

The estimation results of the interaction terms suggest that air quality aggravated ADL disability caused by serious illness, and the interaction terms of serious illness and the concentrations of SO_2_ and NO_2_ were the main factors in the positive promotion effect on ADL disability. The primary reason for this might be that the increase in air pollutants increases the probability of residents suffering from serious illness, thus aggravating the risk of ADL disability.

### Extensive analysis

The effect of air quality on ADL disability has been analysed. Furthermore, to fix the problems of self-selection bias and missing variables in samples, we used control samples and considered two-way fixed effects in a more robust model.

### Bias processing of the self-selection sample

Due to the environmental migration in the process of air pollution, the estimation results are likely biased. To reduce the estimation bias caused by environmental migration, in the sample processing step, a subsample test was conducted for the participants whose residence location and groups did not change. The results are given in Table 7. It becomes clear that SO_2_ had a negative impact on ADL disability at the 10% significance level, NO_2_ had a negative impact on ADL disability at the 5% significance level, and the AQI had a negative impact on ADL disability at the 10% significance level. Therefore, the findings of previous models were robust.

**Table 7:** Effect of air quality on ADL disability of permanent residents

Variable	(1)	(2)	(3)	(4)
lnSO_2_	−0.0684*(0.0362)			
lnNO_2_		−0.0183(0.0544)		
lnPM_10_			−0.1307**(0.0521)	
lnAQI				−0.1368*(0.0772)
Serious illness	0.0563**(0.0279)	0.0570**(0.0279)	0.0586**(0.0279)	0.0477(0.0291)
Depression	0.1265***(0.0256)	0.1255***(0.0256)	0.1268***(0.0256)	0.1261***(0.0267)
Self-reported health	0.0850***(0.0203)	0.0855***(0.0203)	0.0860***(0.0203)	0.0864***(0.0212)
Body disability	−0.6325***(0.0495)	−0.6323***(0.0495)	− 0.6324***(0.0495)	−0.6282***(0.0516)
Physical pain	−0.0342(0.0219)	−0.0349(0.0218)	− 0.0343(0.0219)	−0.0449**(0.0227)
Individual / Year	Yes	Yes	Yes	Yes
/sigma2_u	1.7967***(0.1216)	1.7962***(0.1216)	1.8010***(0.1218)	1.8027***(0.1296)
Pseudo log likelihood	−26,292.693	−26,294.516	−26,291.386	−24,363.95
Observations	25,169	25,169	25,169	25,169

*Abbreviations*: *AQI* Air Quality Index, *SO*_2_ Sulfur Dioxide, *NO*_2_ Nitrogen Dioxide, *PM*_10_ Inhalable Particles

Note: Standard errors are in brackets; *** *p* < 0.01, ** *p* < 0.05, * *p* < 0.1. The control variable results are not listed here

### Treatment of bidirectional fixed effects of panel data

Although the above analysis synchronously controlled for the corresponding individual sociodemographic characteristics and urban environmental characteristics, missing variables might still exist and result in estimation bias. Therefore, we first used a two-way fixed effects model to address the endogeneity problem caused by missing variables. This was referred to by Liu and Hu [17], who viewed classified variables as continuous variables and employed a linear two-way fixed effects model. In this case, ADL disability was considered a continuous variable, and the test results for this model are presented in Table 8. As a result, SO_2_, NO_2_ and the AQI did not show a significant effect on ADL disability. The significance levels of SO_2_ and the AQI were decreased in the fixed effects model, but they were still significant at 15%. PM_10_ had a significant and negative effect on ADL disability at a significance level of 1%, and the significance of PM_10_ was higher than the results of the benchmark model. Therefore, air quality still had a significant impact on ADL disability in the panel two-way fixed effects model, which means that the result was robust.

**Table 8:** Impact of air quality on ADL disability: Based on fixed effects

Variable	(1)	(2)	(3)	(4)
lnSO_2_	−0.0130(0.0095)			
lnNO_2_		−0.0238(0.0153)		
lnPM_10_			−0.0584***(0.0185)	
lnAQI				0.0002(0.0003)
Serious illness	−0.0157***(0.0054)	−0.0155***(0.0054)	− 0.0149***(0.0054)	−0.0155***(0.0054)
Depression	0.0135**(0.0057)	0.0134**(0.0057)	0.0139**(0.0057)	0.0134**(0.0057)
Self-reported health	−0.0071(0.0050)	−0.0070(0.0050)	− 0.0065(0.0050)	−0.0072(0.0050)
Body disability	−0.0651***(0.0119)	−0.0652***(0.0119)	− 0.0658***(0.0119)	−0.0649***(0.0119)
Physical pain	−0.0126***(0.0036)	−0.0129***(0.0036)	− 0.0128***(0.0036)	−0.0128***(0.0036)
Individual / Year	Yes	Yes	Yes	Yes
F Test	5.87	5.89	6.26	5.81
R^2^	0.0127	0.0127	0.0135	0.0126
Observations	26,218	26,218	26,218	26,218

*Abbreviations*: *AQI* Air Quality Index, *SO*_2_ Sulfur Dioxide, *NO*_2_ Nitrogen Dioxide, *PM*_10_ Inhalable Particles

Note: Standard errors are in brackets; *** *p* < 0.01, ** *p* < 0.05, * *p* < 0.1. The control variable results are not listed here

### Instrumental variables

We further adopted the instrumental variable method for endogenous processing. An ordered probit instrumental variable method was selected. According to previous studies, the abundance of regional mineral resources and the proportion of mining industry employees in the total population could be used as instrumental variables of air quality [17]. Therefore, we chose the proportion of mining industry employees in the total population as the proxy variable of regional mineral resources and constructed the two-stage method of IV for the ordered probit model. The results are given in Table 9.

**Table 9:** Estimation results of the IV ordered probit model

Variable	(1)		(2)		(3)		(4)	
	First-stage lnSO_2_	Second-stage ADL	First-stage lnNO_2_	Second-stage ADL	First-stage lnPM_10_	Second-stage ADL	First-stage lnAQI	Second-stage ADL
Mineral endowment	18.0074***(0.3361)		6.4223*** (0.2497)		7.9056*** (0.2517)		1.8217*** (0.1610)	
lnSO_2_		−0.2086***						
		(0.0481)						
lnNO_2_				−0.5079***				
				(0.1249)				
lnPM_10_						−0.4492***		
						(0.1048)		
lnAQI								−1.8083***
								(0.3437)
Serious illness		0.0195*		0.0192*		0.0225**		0.0201**
		(0.0106)		(0.0104)		(0.0105)		(0.0095)
Depression		0.0744***		0.0722***		0.0896***		0.0811***
		(0.0106)		(0.0104)		(0.0106)		(0.0105)
Self-reported health		0.0366***		0.0360***		0.0331***		0.0295***
		(0.0089)		(0.0087)		(0.0088)		(0.0080)
Body disability		−0.3027***		−0.2968***		−0.2951***		−0.2653***
		(0.0163)		(0.0164)		(0.0163)		(0.0210)
Physical pain		−0.0214**		−0.0210**		−0.0243***		−0.0216***
		(0.0084)		(0.0082)		(0.0083)		(0.0076)
Individual / Year		YES		YES		YES		YES
|*α*/*β*|		10.6974		26.4531		19.9644		89.9652
First stage F value	2870.47		661.23		976.54		128.13	128.13
Adjust R^2^	0.0736		0.0180		0.0263		0.0035	0.0035
Observations	26,218	26,218	26,218	26,218	26,218	26,218	26,218	26,218

*Abbreviations*: *AQI* Air Quality Index, *SO*_2_ Sulfur Dioxide, *NO*_2_ Nitrogen Dioxide, *PM*_10_ Inhalable Particles

Note: Standard errors are in brackets; *** *p* < 0.01, ** *p* < 0.05, * *p* < 0.1. The control variable results are not listed here

From the results of the first-stage test in Models (1) to (3), mineral resources have a significant and positive effect on air quality. The validity test of instrumental variables shows that the F value in the first stage is significantly greater than 10, indicating that the problem of weak instrumental variables did not exist. In other words, the selection of instrumental variables was effective. The results of second-stage tests in Models (1) to (3) show that air quality had a significant and negative impact on residents’ ADL disability at the significance level of 1%, which further demonstrates that the results of this study are robust. The results for Model (4) suggest that the AQI still had a significant and negative effect on ADL disability. This further proves that poor air quality significantly aggravates ADL disability. In addition, it can be seen from Model (4) that to keep ADL disability unchanged, ADL disability caused by a 1% increase in the AQI requires an 89.9652% reduction in serious disease to compensate for ADL damage. This means that the reduction amount of ADL disability brought by a 1-unit improvement in air quality equals the amount caused by a 89.9652-unit decrease in severe illness.

## Discussion

The air quality index at a certain time point is a comprehensive indicator of pollutant accumulation, which is also an accurate reflection of air pollution at a specific time point. Thus, this study takes the annual average value of the AQI in a region as the proxy index to reflect long-term air pollution. At the same time, a multidimensional measurement of ADL could be established by dividing the disability level into five levels: health, mild disability, moderate disability, severe disability and serious disability [6]. Based on this, this study empirically tests the impact of the AQI on residents’ ADL disability. The results demonstrate that air quality has a significant impact on residents’ ADL disability, which is mainly manifested by the health reduction effect and increasing effect on ADL disability. Compared with existing studies, this study breaks the mould by exploring the impact of the AQI on residents’ ADL disability from the perspective of air pollution and enriches the research perspective of the social cost of air pollution. In addition, this study uses the average value of the AQI as the proxy variable of air quality, which can empirically reflect the impact of long-term exposure to air pollution on residents’ ADL disability.

Most existing studies have investigated the impact of air pollution from the perspective of social risk cost. For example, several scholars have estimated the impact of air pollution on residents’ health outcomes [11–15]. The indicators of health include changes in individual health level or changes in the incidence of disease and the incidence rate of diseases in the whole region (such as lung cancer mortality or respiratory disease mortality per 10,000 people) [17–21], as well as the increase in the cost of treatment due to air pollution, which indicates the social cost of air pollution [22, 23]. Therefore, the reliability of the conclusions of this study is a further expansion of the scope of existing findings. First, this study not only investigated the direct health outcome of air pollution but also investigated the changing paths of residents’ health influenced by air pollution, for instance, by analysing the change in the prevalence of major diseases; thus, discussion of the changing path of residents’ ADL disability under the influence of air pollution could be extended. This study also empirically reveals the theoretical basis for the social governance of residents’ ADL disability and the optimization of long-term care insurance. Specifically, the findings of this study provide insights into environmental governance of residents with ADL disability [47–49]. For example, investigating the changes in the disability rate and factors of disability risk of local residents and the effective regulations of air pollution could be undertaken for environmental governance. In this study, this is mainly explained by investigating the impact of different pollutants on residents’ ADL disability. The results of this study clarify that the concentrations of SO_2_ and PM_10_ pollutants are the main elements affecting residents’ ADL disability. In addition, there are significant group differences in the impact of air quality on ADL disability. For example, air quality has a more significant impact on the ADL disability of elderly residents aged 60 and above, female residents, residents in regions with low economic levels, and residents in areas without pilot long-term insurance. Additionally, the negative effect of air pollution is stronger on these groups. These findings demonstrate that the health damage effect of air pollution can be effectively reduced after the implementation of effective social policy intervention. Furthermore, few studies or practices have calculated the cost of disability treatment caused by pollution or the cost borne by the entire society. This is also one of the main innovations of this study. Ultimately, we should not only realize the significant impact of air pollution on ADL disability but also consider the differences between different groups and take the most effective measures to control air pollution and reduce its long-term social cost, that is, the long-term care cost of treating disabled residents.

Despite the practical significance of the findings of this study, especially the results of the analysis, which have been proven valid after a series of robustness tests and endogenous treatments, this study still has some limitations. First, the sample of this study mainly comes from 122 cities in China, but the sample is limited to people over 45 years old and generally excludes those under 45 years old, which may affect the applicability and reliability of the conclusions to a certain extent. Second, as one of the most important purposes of this study, ADL disability caused by air pollution and the cost of disability treatment are the main focus of this study. However, due to the complexity of factors that cause disability and the indeterminacy of actual nursing costs, this study was not able to measure the social cost of ADL disability caused by current air pollution completely and accurately, which would also be an important direction for further research. By gradually fixing the above problems, we can further clarify the marginal effect and social cost of air pollution governance and theoretically provide important support for optimizing regional policy for disability prevention and long-term care service security. The main advantages of this study are that it not only explores the direction of the impact of the AQI on residents’ ADL disability but also investigates the specific effect of the AQI on residents’ ADL disability, in addition to the changing trend of the long-term ADL disability rate caused by the joint influences of the AQI and the rate of serious disease. Thus, the logical relationship and mutual effects between natural environmental factors (AQIs) and individual health characteristic factors (rate of serious disease) are entangled.

## Conclusions

This study used tracking data from the CHARLS database from 2015 and 2018 to construct panel data for investigating the impact of air quality on ADL disability and its marginal effect. The results show that air quality has a significant impact on ADL disability, and the main impacts were from the concentrations of SO_2_ and PM_10_. Second, in terms of the marginal effect, the main effects of air quality on ADL disability appear to have a positive effect on disability increment, and it also shows that air quality plays a leading role in the negative effect of health. Moreover, it was revealed that air quality has a more significant impact on the ADL disability of residents aged 60 years and above, female residents, residents with poor economic status and residents in areas without LTCI. The results of the interaction between air quality and serious illness showed that air quality worsened the impact of serious illness on ADL disability. Finally, we confirmed the robustness of our findings by controlling subsamples and using two-way fixed effects and instrumental variables.

Our findings are also strongly relevant to policy decisions. First, social and economic development should be “environmentally friendly” and should not only consider the short-term increase in GDP but also consider the basic quality of life of local residents, especially the health of vulnerable groups such as the elderly population and those living in economically underdeveloped areas. Second, controlling air pollution should be prioritized. The impact of air quality on the natural environment of a country or region has been remarkable, and its impact on the health of individuals is also gradually being recognized. The increase in ADL disability caused by the increase in the incidence of individual serious illness influenced by air quality also indicates that the social cost of environmental pollution is increasing. Third, when investigating ADL disability in theory, we should not only pay attention to the causes of disability from the perspective of traditional medicine or socioeconomic environments but also note the influences of ecological environment changes and the negative impacts of changes in air quality. Therefore, intervention policies could be implemented to prevent ADL disability and improve quality of life.

## Data Availability

The data that support the findings of this study are openly available at the following URL/DOI: http://charls.pku.edu.cn/

## Abbreviations

LTCI: Long-term Care Insurance
CHARLS: China Health and Retirement Longitudinal Study
AQI: Air Quality Index
HAQI: Health Risk-based AQI
API: Air Pollution Index
PM_2.5_: Fine Particulate Matter
PM_10_: Inhalable Particles
O_3_: Ozone
CO: Carbon Monoxide
SO_2_: Sulfur Dioxide
NO_2_: Nitrogen Dioxide
GDP: Gross Domestic Product
